# Multiple competing pathways for chemical reaction: drastic reaction shortcut for the self-catalytic double-helix formation of helicene oligomers†
†Electronic supplementary information (ESI) available. See DOI: 10.1039/c6sc01893a



**DOI:** 10.1039/c6sc01893a

**Published:** 2016-10-14

**Authors:** Yo Kushida, Nozomi Saito, Masanori Shigeno, Masahiko Yamaguchi

**Affiliations:** a Department of Organic Chemistry , Graduate School of Pharmaceutical Sciences , Tohoku University , Aoba , Sendai , 980-8578 , Japan . Email: yama@m.tohoku.ac.jp

## Abstract

Competition among multiple pathways in a chemical reaction exhibits notable kinetic phenomena, particularly when amplification by self-catalysis is involved. A pseudoenantiomeric 1 : 1 mixture of an aminomethylene helicene (*P*)-tetramer and an (*M*)-pentamer formed enantiomeric hetero-double helices **B** and **C** in solution when random coil **A** was cooled. When a solution of **A** at 70 °C was directly cooled to 25 °C, the **A**-to-**B** reaction was predominant, then **B** was slowly converted to **C** over 60 h. The slow conversion in the **A**-to-**B**-to-**C** reaction was due to the formation of the hetero-double helix **B**, which was an off-pathway intermediate, and the slow **B**-to-**C** conversion. In contrast, when a solution of **A** at 70 °C was snap-cooled to –25 °C before then maintaining the solution at 25 °C, the **A**-to-**C** reaction predominated, and the formation of **C** was complete within 4 h. The reactions involve competition between the self-catalytic **A**-to-**B** and **A**-to-**C** pathways, where **B** and **C** catalyze the **A**-to-**B** and **A**-to-**C** reactions, respectively. Subtle differences in the initial states generated by thermal pretreatment were amplified by the self-catalytic process, which resulted in a drastic reaction shortcut.

## Abstract 2

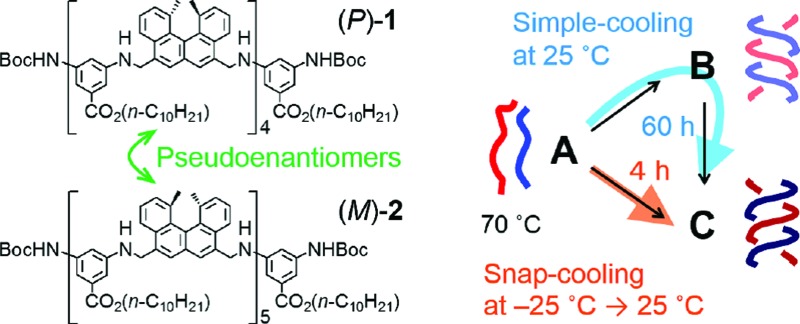
A drastic reaction shortcut: thermal history determines the selection of competing pathways and reaction time for self-catalytic hetero-double-helix formation.

## Introduction

**Fig. 1:**
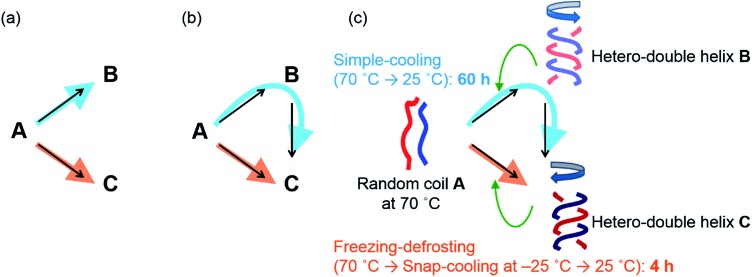
Competitive multiple pathways in chemical reactions providing (a) different products **B** and **C** and (b) an identical product **C**. (c) Multiple competing pathways in the self-catalytic reaction of the (*P*)-**1**/(*M*)-**2** system resulting in a drastic reaction shortcut. It takes about 60 h for the **A**-to-**B**-to-**C** reaction to complete at 25 °C under simple-cooling (70 °C to 25 °C) conditions, while the **A**-to-**C** reaction at 25 °C is completed in 4 h under freezing–defrosting (70 °C followed by snap-cooling at –25 °C then heating to 25 °C) conditions, which are governed by the thermal pretreatment. Green arrows indicate self-catalysis.

Chemical reactions are interesting phenomena of molecular structural changes in nature, including biological systems, and the understanding of their properties and mechanisms is an important subject in chemistry. Chemical reactions, however, are extremely complex because of nonequilibrium thermodynamics, and kinetically unusual phenomena occur during transitions from the metastable states of substrate molecules to the stable states of product molecules.^1^ Multiple pathways (Fig. 1a and b) in a chemical reaction involve many transition states and intermediates, and the number of molecules following each pathway is also an important factor.^2–6^ Studies of such competing multiple pathways are essential for understanding chemical reactions. This paper treats chemical reactions in a broader sense, which includes rearrangement of the noncovalent as well as covalent bonds.

Multiple competing pathways (Fig. 1a and b) in a chemical reaction are intriguing because reaction pathways can change considerably depending on conditions, as has been observed in the formation of amyloid fibrils during aggregation.^2^ Such phenomena can be enhanced particularly when an amplification mechanism is involved, such as self-catalysis, in which product molecules catalyze the reaction of substrate molecules to become product molecules.^7^ Therefore, slight differences in the initial states can greatly affect the competition of the reaction pathways. Here, we report a drastic change in reaction time for the formation of hetero-double helices^8–10^ as a result of switching competing pathways (Fig. 1c). It is noted that subtle differences in the initial states generated by thermal pretreatment are amplified by self-catalysis and that a drastic reaction shortcut occurs in dilute solution at the molecular level. It is noteworthy that both pathways give an identical product from an identical starting material (Fig. 1b and c) but take different times.

**Fig. 2:**
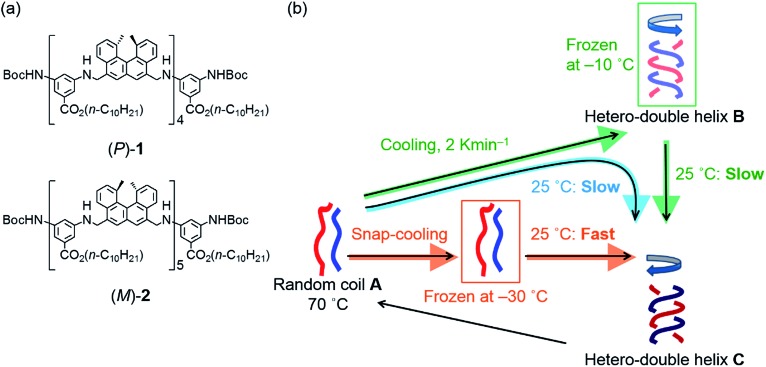
(a) Chemical structures of aminomethylenehelicene oligomers (*P*)-**1** and (*M*)-**2**. (b) Schematic presentation of random coil **A**, enantiomeric hetero-double helices **B** and **C**, and the three-state one-directional structural change. The **A**-to-**B**-to-**C** reaction under the simple-cooling condition is shown with a blue arrow; the **A**-to-**B**-to-**C** reaction *via* **B** trapped by cooling from 70 to –10 °C at a rate of 2 K min^–1^ is shown with green arrows; the **A**-to-**C** reaction under the freezing–defrosting condition is shown with orange arrows.

Previously, we reported the structural change in a pseudoenantiomeric 1 : 1 mixture of the aminomethylenehelicene^11,12^ (*P*)-tetramer (*P*)-**1** and the (*M*)-pentamer (*M*)-**2** (ref. 13) (Fig. 2a) in solution between the random coil **A**, the hetero-double helix **B**, and the hetero-double helix **C** (Fig. 2b).^14^ The structures **B** and **C** are very close to being enantiomers, but are not energetically identical, because **B** and **C** are derived from the (*P*)-tetramer (*P*)-**1** and the (*M*)-pentamer (*M*)-**2**, which are enantiomeric in terms of the helicene absolute configuration but differ in the number of helicenes. Cooling random coil **A** from 70 °C to 25 °C provided hetero-double helix **B**. Then, **B** was slowly converted to **C** by maintaining the solution at 25 °C (simple-cooling conditions) (Fig. 2b, blue arrow). The **A**-to-**B**-to-**C**-to-**A** three-state one-directional molecular structural change occurred with thermal hysteresis, in which the involvement of a self-catalytic (autocatalytic) mechanism was suggested. It was also found that the metastable double helix **B** could be trapped by decreasing the temperature from 70 to –10 °C at a rate of 2 K min^–1^, and a slow conversion to **C** occurred when the solution at –10 °C was warmed to 25 °C (Fig. 2b, green arrows). In another experiment, **A** was trapped by the snap-cooling of the solution from 70 to –30 °C, and a relatively fast conversion to **C** occurred upon warming to 25 °C (freezing–defrosting condition) (Fig. 2b, orange arrow).

In this study, we turned our attention to the marked difference in reaction time for the conversion starting from **A** to produce **C** depending on different thermal pretreatments, which were examined in detail under the freezing–defrosting and simple-cooling conditions. A drastic reaction shortcut was observed because of competing self-catalytic pathways starting from random coil **A** to produce hetero-double helix **B** or **C** depending on the thermal pretreatment: cooling of random coil **A** at 70 °C to 25 °C and then maintaining the solution at 25 °C (simple-cooling) gave double helix **C** after 60 h *via* double helix **B**; snap-cooling of **A** at 70 °C to –25 °C and then maintaining the solution at 25 °C (freezing–defrosting) gave **C** within 4 h (Fig. 1c).

A notable feature of this system shown in this work is the involvement of competing self-catalytic reactions: **B** and **C** are formed from substrate **A**, and **B** and **C** catalyse the reaction to form **B** and **C**, respectively (Fig. 1c). Therefore, the balance between the two reaction pathways and the product distribution [**B**]/[**C**] is highly sensitive to subtle differences in the initial states. Another notable feature is that this system involves molecular-level phenomena, not polymolecular self-assembly.^5^ It should also be noted that a drastic reaction shortcut occurs reversibly in a closed system, which is driven only by thermal pretreatment without the addition of a catalyst, a change in concentration, or seeding:^5^ the thermal history results in a reaction shortcut.

## Results and discussion

### Drastic reaction shortcut

**Fig. 3:**
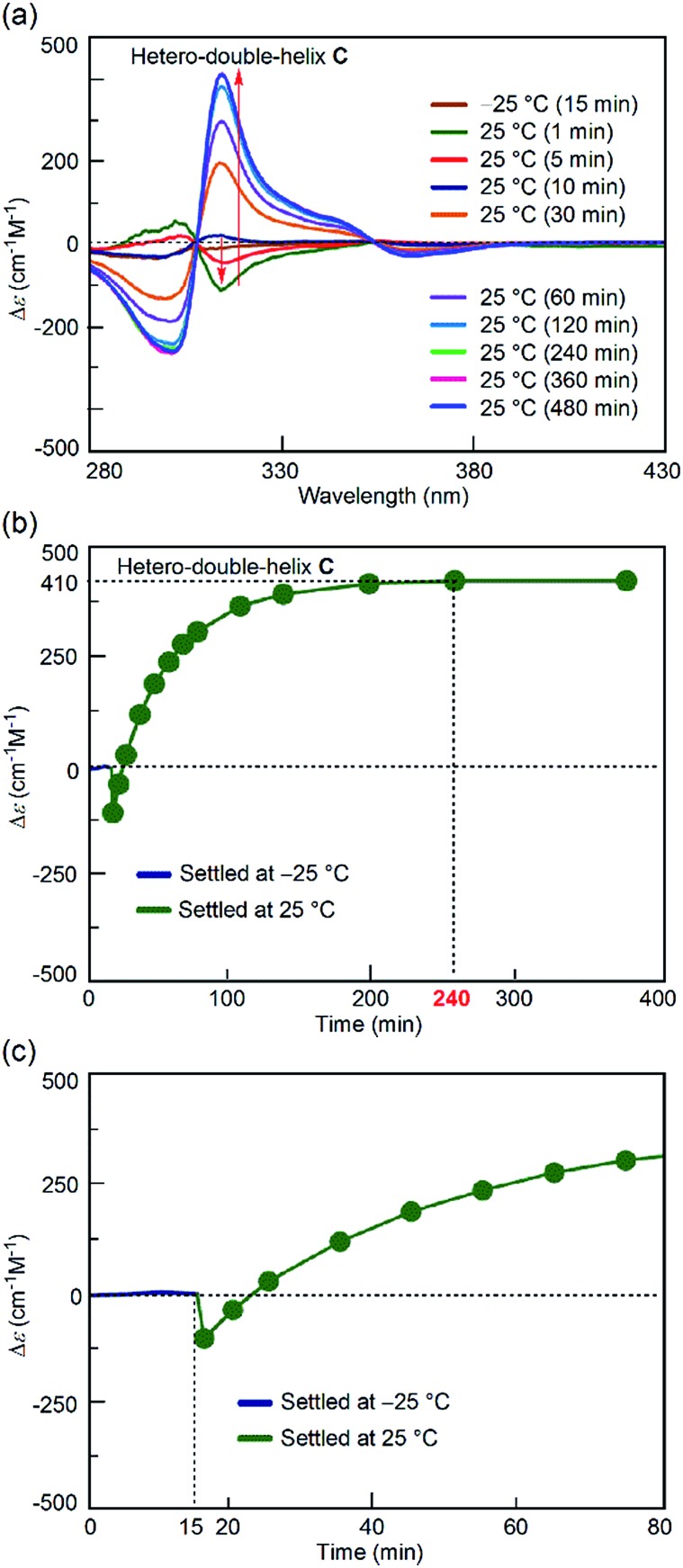
(a) CD spectra and (b) Δ*ε* (315 nm)/time profiles of 1 : 1 (*P*)-**1**/(*M*)-**2** mixtures in fluorobenzene (total concentration, 5.0 × 10^–4^ M) showing structural changes from the random coil **A** at 70 °C to the hetero-double helix **C** at 25 °C under the freezing–defrosting/rapid heating conditions. (c) A magnification of (b).

To probe the origin of the drastic reaction shortcut, the freezing–defrosting experiment was conducted and studied in detail. Rapid heating was conducted for “defrosting”, as described below. A 1 : 1 mixture of (*P*)-**1** and (*M*)-**2** in fluorobenzene (total concentration, 5.0 × 10^–4^ M) was heated to 70 °C to prepare a solution of S-random-coil **A**, in which essentially all the molecules were dissociated and existed as random coil **A**. The solution of **A** was snap-cooled to –25 °C by immersing it in a cooled bath at –35 °C and then was maintained at –25 °C for 15 min (see the ESI† for details), during which time no change was observed in the CD spectrum with a weak Cotton effect (Fig. 3a, brown line). The solution was rapidly heated to 25 °C (freezing–defrosting/rapid conditions) for 1 to 1.5 min. The CD intensity of the negative Cotton effect at 315 nm increased after 1 min (Fig. 3a, dark green line), suggesting the formation of a small amount of the hetero-double helix **B**.^14^ Then, the intensity decreased, became positive at 10 min, and continued to increase for 4 h (240 min), eventually giving the spectrum of the hetero-double helix **C**.^14^ The spectrum remained unchanged after 8 h, which indicated an equilibrium (Fig. 3a).

The structural change was monitored by the Δ*ε* value at 315 nm (Fig. 3b). A Δ*ε* value of 0 cm^–1^ M^–1^ was obtained immediately after heating the “frozen” solution of **A** obtained by snap-cooling at –25 °C rapidly to 25 °C, and maintaining the solution at 25 °C (freezing–defrosting/rapid conditions). The value decreased to –105 cm^–1^ M^–1^ after 1 min, which suggested the formation of a small amount of the hetero-double helix **B**, then Δ*ε* increased to +110 cm^–1^ M^–1^ after 20 min and reached +410 cm^–1^ M^–1^ after 4 h. It was comparable to the S-hetero-double-helix **C** state giving the Δ*ε* value of +440 cm^–1^ M^–1^ determined by our previous experiments,^14^ where essentially all the molecules formed the hetero-double helix **C**. Predominant formation of hetero-double helix **C** after 4 h was indicated.

A slower heating experiment was also conducted (Fig. S1†), where the temperature of a “frozen” solution of **A** obtained by snap-cooling at –25 °C was increased to 25 °C at a constant rate of 2 K min^–1^ (freezing–defrosting/constant-rate experiment). Essentially the same results were obtained in the rapid (Fig. 3b and c) and constant-rate (Fig. S1†) heating experiments, and the heating rate did not affect the rate of the formation of **C**.

**Fig. 4:**
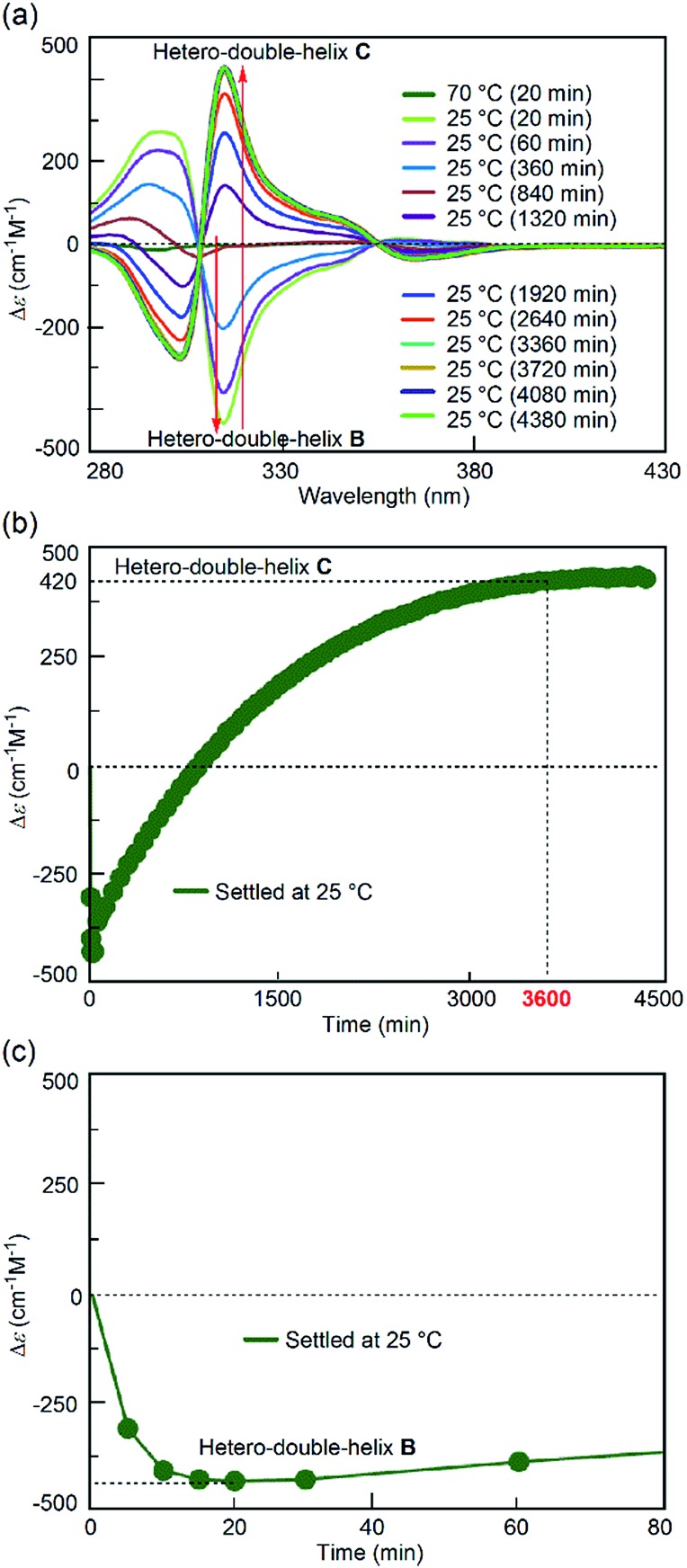
(a) CD spectra and (b) Δ*ε* (315 nm)/time profiles of 1 : 1 (*P*)-**1**/(*M*)-**2** mixtures in fluorobenzene (total concentration, 5.0 × 10^–4^ M) showing structural changes from the random coil **A** at 70 °C to the hetero-double helix **C** at 25 °C under the simple-cooling/rapid heating conditions. (c) A magnification of (b).

For comparison, the simple-cooling experiments under rapid cooling and constant-rate cooling conditions were conducted using a 1 : 1 mixture of (*P*)-**1**/(*M*)-**2** in fluorobenzene. A solution of the S-random-coil **A** (total concentration, 5.0 × 10^–4^ M) at 70 °C was prepared and rapidly cooled to 25 °C for 1 to 2 min without the “freezing” process (simple-cooling/rapid experiment, see the ESI† for details). The Δ*ε* at 315 nm first decreased and gave the spectrum of the hetero-double helix **B** (Fig. 4a, light green line). The Δ*ε* value after 20 min was –420 cm^–1^ M^–1^ (Fig. 4c), which coincided with that of the S-hetero-double-helix **B** (–430 cm^–1^ M^–1^), where all the molecules formed **B**.^14^ Then, the Cotton effect inverted and reached an equilibrium by maintaining the solution at 25 °C for 56 h (3360 min) (Fig. 4a), giving a Δ*ε* of +420 cm^–1^ M^–1^ at 315 nm (Fig. 4b), which is close to the Δ*ε* of the S-hetero-double-helix **C**. An experiment of cooling at a rate of 2 K min^–1^ from 70 to 25 °C (simple-cooling/constant-rate experiment) provided the same result (Fig. S2†).

### Two reaction pathways

The structures of the (*P*)-**1**/(*M*)-**2** mixtures obtained at the initial state of 25 °C were compared in the freezing–defrosting/rapid and simple-cooling/rapid experiments. In both experiments, essentially the same CD spectra were obtained within 60 seconds (Fig. S3 and S4†), which indicated the formation of random coil **A**. The ^1^H-NMR spectra obtained under each condition coincided (Fig. S5†). Dynamic light scattering (DLS) analysis confirmed the presence of dispersed states at 25 °C for both conditions (Fig. S6†), and not formation of an ununiform polymolecular assembly, in accordance with the experiments using DLS and a Job plot in the S-hetero-double-helix **B** and **C** state in our previous study.^14^ Thus, the spectroscopically identical state **A** was obtained at 25 °C after both the freezing–defrosting/rapid and simple-cooling/rapid pretreatment.

Different reactions occurred after 60 seconds: in the freezing–defrosting/rapid experiment, a decrease of Δ*ε* at 315 nm indicated the formation of a small amount of **B**, which was followed by the increase of the Δ*ε* value after 280 s (Fig. S3†) indicating the formation of hetero-double helix **C**. The **A**-to-**C** and **A**-to-**B** reactions competed with each other. It was noted that **C** was directly formed from **A** but not from **B** under this condition, as indicated by the consistent isosbestic points in the CD spectra (Fig. 3a). A small amount of **B** formed at the early stage likely underwent a slow transition to **C**. In the simple-cooling/rapid experiment, the Δ*ε* value kept decreasing until it reached the S-hetero-double-helix **B** state (Fig. S4† and 4), where the **A**-to-**B**-to-**C** reaction was predominant.

**Fig. 5:**
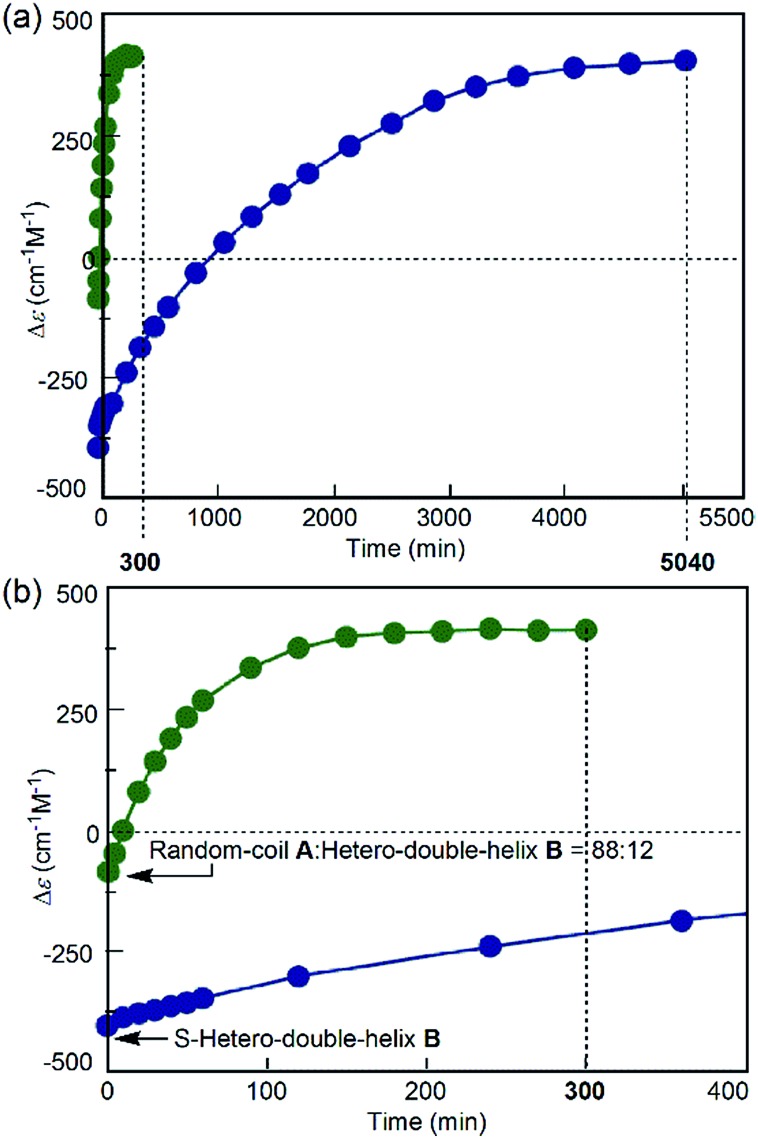
(a) Δ*ε* (315 nm)/time profiles of 1 : 1 (*P*)-**1**/(*M*)-**2** mixtures in fluorobenzene (total concentration, 5.0 × 10^–4^ M) showing structural changes from the random coil **A** at 70 °C to the hetero-double helix **C** at 25 °C after cooling at –10 °C by snap-cooling (green circles) and at the rate of 2 K min^–1^ (blue circles). (b) A magnification of (a). Lines are drawn between the points.

The direct **A**-to-**C** conversion under the freezing–defrosting/rapid conditions was further confirmed by comparing the **A**-to-**C** and **A**-to-**B** reactions under the following conditions (Fig. 5). The (*P*)-**1**/(*M*)-**2** solution (total concentration, 5.0 × 10^–4^ M) in fluorobenzene was heated to 70 °C, snap-cooled to –10 °C, which resulted in a Δ*ε* value of –88 cm^–1^ M^–1^ immediately after cooling at –10 °C (Fig. 5, green circle, *t* = 0). Then, **C** was formed by rapidly heating to 25 °C and maintaining the temperature for 300 min (Fig. 5, green circles; Fig. S7a†). In order to compare the **A**-to-**C** pathway with the **A**-to-**B** pathway, the (*P*)-**1**/(*M*)-**2** solution (total concentration, 5.0 × 10^–4^ M) was heated at 70 °C and cooled to –10 °C at a constant rate of 2 K min^–1^ to form **B**.^14^ The Δ*ε* value of –430 cm^–1^ M^–1^ at –10 °C (Fig. 5, blue circle, *t* = 0) confirmed the predominant formation of **B** (Fig. S7b†). Then, the solution was rapidly heated to 25 °C and maintained at that temperature, where **B** was slowly converted to **C** for 5040 min (Fig. 5, blue circles; Fig. S7b†). Formation of **C** was very slow once **B** became predominant, and the hetero-double helix **B** is an off-pathway intermediate, which hampers the **A**-to-**C** reaction.^2c,3,5^ The considerable difference in the reaction time between these results indicated the presence of different reaction pathways: the fast **A**-to-**C** reaction under the freezing–defrosting conditions and the slow **A**-to-**B**-to-**C** reaction under the simple-cooling conditions.

The results at lower concentrations of 2.5 × 10^–4^ M (Fig. S8–S12†) supported the off-pathway mechanism. Although a tendency similar to that at 5.0 × 10^–4^ M was observed at a concentration of 2.5 × 10^–4^ M (Fig. S11†), it was noted that the formation of **C** under freezing–defrosting/rapid conditions was slightly faster at 2.5 × 10^–4^ M (Fig. S9†) than at 5 × 10^–4^ M (Fig. S13†). Related phenomena have been observed in self-assembly with complex pathways involving off-pathway mechanisms.^2c,3d,5a^

**Fig. 6:**
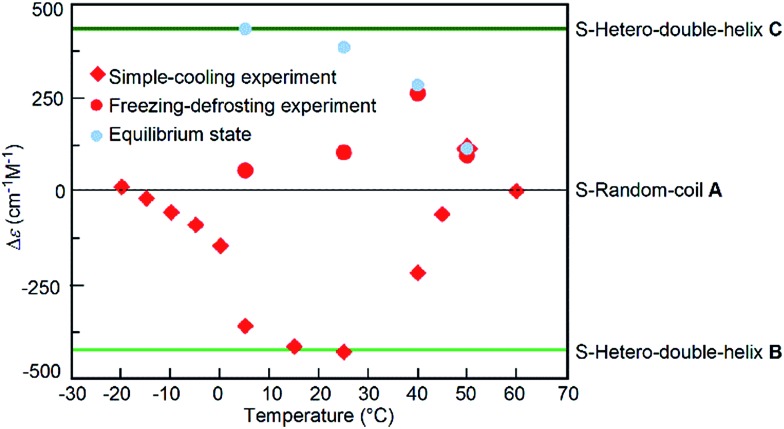
Δ*ε* (315 nm)/temperature profiles of 1 : 1 (*P*)-**1**/(*M*)-**2** mixtures in fluorobenzene (total concentration, 5.0 × 10^–4^ M), which were obtained by plotting the Δ*ε* values reached after 20 min periods under the freezing–defrosting/rapid conditions (red circles) and those after 20–30 min under the simple-cooling/rapid conditions (red squares). The Δ*ε* values at equilibrium are also shown (blue circles). Data were obtained from Fig. S13 and S14.† The Δ*ε* values of S-random-coil **A**, S-hetero-double-helices **B** and **C**, and at the equilibrium between 5 and 50 °C were obtained in our previous work.^14^

The freezing–defrosting/rapid experiments were conducted at different temperatures of 5, 25, 40, and 50 °C, which were obtained by rapidly heating from –25 °C, and the Δ*ε*/time profiles were obtained (Fig. S13†). At 5 °C, a Δ*ε* of +56 cm^–1^ M^–1^ was observed after maintaining the solution at that temperature for 20 min, and +440 cm^–1^ M^–1^ was reached after 300 min; equilibrium at 5 °C also resulted in a Δ*ε* of +440 cm^–1^ M^–1^ (Fig. S13†).^14^ A slight sigmoidal curve was obtained. A similar result was obtained by maintaining the solution at 25 °C. At 40 °C and 50 °C, the reactions were faster. The freezing–defrosting/rapid experiments at different temperatures are summarized by plotting the Δ*ε* values obtained after 20 min of heating against the temperature in order to analyse the kinetic process (Fig. 6, red circles). For comparison, the plots of Δ*ε* values at the equilibrium of the **A**-to-**C** reaction which were obtained by maintaining the mixtures for a sufficiently long time at each temperature are also shown (Fig. 6, blue circles; Fig. S13†).^14^ The Δ*ε* values at 20 min in the freezing–defrosting experiment consistently appeared in the positive region of the Δ*ε* values, indicating the predominance of the **A**-to-**C** reaction over the **A**-to-**B** reaction.

For comparison, experiments at different temperatures were conducted under simple-cooling/rapid conditions between –20 and 50 °C (Fig. S14†), and the Δ*ε* values between –20 and 40 °C at 20–30 min were plotted against temperature (Fig. 6, red squares). At these temperatures, negative Δ*ε* values were observed, which indicated the predominant occurrence of the **A**-to-**B** reaction. Below 0 °C, the Δ*ε* values were between 0 and –100 cm^–1^ M^–1^, indicating small changes after maintaining the mixture at the given temperature, which were due to the slow **A**-to-**B** reaction. The Δ*ε* values at temperatures between 5 and 25 °C were close to that of S-hetero-double-helix **B**:–430 cm^–1^ M^–1^. At 40 °C, Δ*ε* increased slightly to –220 cm^–1^ M^–1^, but was still negative. Notably at 50 °C, a slow increase of the Δ*ε* occurred in the simple-cooling/rapid experiment and a positive Δ*ε* of +80 cm^–1^ M^–1^ was obtained at 600 min (Fig. S14,† brown circles), which indicated the predominant occurrence of the **A**-to-**C** reaction over the **A**-to-**B** reaction at 50 °C. Pathway competition between the **A**-to-**C** and **A**-to-**B** reactions is sensitive to temperature, and a sharp switching occurred with small temperature changes at approximately 40 °C.

**Fig. 7:**
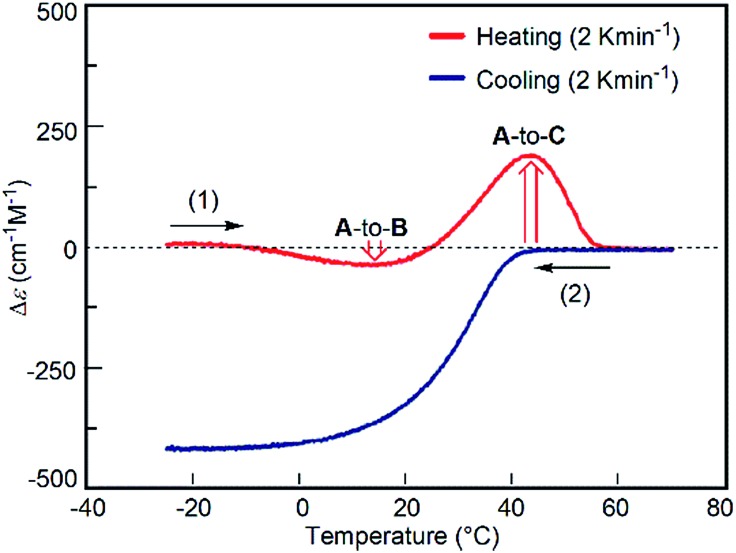
Δ*ε* (315 nm)/time profiles of 1 : 1 (*P*)-**1**/(*M*)-**2** mixtures in fluorobenzene (total concentration, 5.0 × 10^–4^ M) showing the constant-rate temperature change experiment. The “frozen” solution obtained by cooling the solution at 70 °C to –25 °C was (1) heated at a constant rate of 2 K min^–1^ to 70 °C, which was followed by (2) cooling to –25 °C at the same rate. Downward and upward red arrows show the preference of the **A**-to-**B** and **A**-to-**C** reactions, respectively.

The predominant **A**-to-**C** reaction at 50 °C was also observed under the freezing–defrosting/constant-rate conditions (see ESI,† section 7) starting from –25 °C (Fig. 7). The “frozen” solution obtained by cooling the solution at 70 °C to –25 °C was (1) heated at a constant rate of 2 K min^–1^ to 70 °C, which was followed by (2) cooling to –25 °C at the same rate. A slight drop was observed at 15–25 °C upon heating, which indicated a small preference for the **A**-to-**B** reaction. Acceleration of the **A**-to-**C** reaction was noted by the upward rise of the curve at 40–50 °C. When **A** at 70 °C was cooled at the constant rate of 2 K min^–1^, **B** was formed (Fig. 7), which confirmed the reversible nature of the simple-cooling experiments to form **B** from **A**.

To summarize the above results in the pseudoenantiomeric (*P*)-**1**/(*M*)-**2** system, the **A**-to-**C** reaction was predominant under freezing–defrosting conditions, and was complete within 4 h (Fig. 1c, orange arrow). On the other hand, the **A**-to-**B** reaction was predominant under simple-cooling conditions, and was converted to the hetero-double helix **C** after 60 h (Fig. 1c, blue arrow). Random coil **A** was first converted to the metastable hetero-double helix **B**, which was slowly converted to the thermodynamically most stable hetero-double helix **C**. The hetero-double helix **B** acts as an off-pathway intermediate, which hampers the **A**-to-**C** reaction. Differences in the initial states at 25 °C generated by the different thermal pretreatments resulted in the sharp switch of the reaction pathway, the **A**-to-**C** or **A**-to-**B**-to-**C** reaction, and the drastic reaction shortcut occurred, due to amplification by self-catalysis as will be discussed below. Note that **C** is reproducibly obtained under the freezing–defrosting conditions in the (*P*)-**1**/(*M*)-**2** system, and stochastic phenomena to form **B** or **C** do not occur, unlike the chiral symmetry breaking.^15^

### Involvement of self-catalysis

The following experiments confirmed the involvement of self-catalysis in the **A**-to-**C** and **A**-to-**B** reactions. First the self-catalytic nature of the **A**-to-**C** reaction was determined. Slightly sigmoidal kinetics, being consistent with self-catalysis in the **A**-to-**C** reaction, were observed at 5 °C (Fig. S15, S9, and S13†), in which the initial rate was low and later high.^7a,c,f,16^ A seeding experiment confirmed self-catalysis. (*P*)-**1**/(*M*)-**2** in fluorobenzene (total concentration, 5.0 × 10^–4^ M) at 40 °C containing **C** was prepared by cooling the solution of S-random-coil **A** from 70 to 25 °C and maintaining the mixture at that temperature for 3–4 days (see the ESI† for details). The solution of **C** was then heated at 40 °C for 100 min, during which time no change was observed (Fig. S16†). The solution of **A** at 70 °C was rapidly cooled to 40 °C, and the same volume of the above solution of **C** was added within 5 s (Fig. S17a†). A Δ*ε* of +200 cm^–1^ M^–1^ was obtained after 1 min, which increased to +250 cm^–1^ M^–1^ after 5 min and to +290 cm^–1^ M^–1^ after 60 min, which was identified as the equilibrium position for **C** with +290 cm^–1^ M^–1^ at 40 °C (Fig. S17b,† red circles).^14^ The results are compared with those of the experiment without adding **C**: a solution of random coil **A** (concentration 5.0 × 10^–4^ M) was prepared by rapidly cooling the S-random-coil **A** solution from 70 °C to 40 °C; **B** began to form, and a Δ*ε* of –200 cm^–1^ M^–1^ was reached after 20 min (Fig. S17,† orange circles). Thus, seeding the **A** solution with the **C** solution accelerated the **A**-to-**C** reaction and inhibited the **A**-to-**B** reaction. The result is consistent with the involvement of self-catalysis in the **A**-to-**C** reaction. The seeding experiments were also conducted at the ratios of **A** : **C** = 2 : 1 and 3 : 1, and the **A**-to-**C** reaction proceeded (Fig. S17b†).

The involvement of self-catalysis in the **A**-to-**B** reaction was previously suggested on the basis of molecular thermal hysteresis.^14^ The sigmoidal nature of the Δ*ε*/time profile at various temperatures supported the involvement of self-catalysis (Fig. S10†). A seeding experiment was conducted by adding the solution of **B** to the solution of **A** in a 1 : 1 ratio either at 45 °C or 25 °C (see the ESI† for details), which showed an initial increase in **B** (Fig. S18†). In contrast, the control experiments adding no **B** but only the solvent (Fig. S19d†) or the experiments adding **B** to a solution of **B** (Fig. S19e†) showed no sigmoidal change in the Δ*ε*. These results indicated the involvement of self-catalysis in the **A**-to-**B** reaction as well as in the **A**-to-**C** reaction.

Although the molecular mechanism of self-catalysis is not clear at this stage, we presume the formation of a trimolecular complex as discussed in our previous work on the formation of a homo-double-helix by sulfoneamidohelicene oligomers.^17^ A hetero-double helix **B** or **C** works as a template and converts two molecules of random coil **A** to **B** or **C**.^17*b*^

## Conclusions

In summary, a pseudoenantiomeric 1 : 1 mixture of the aminomethylene helicene (*P*)-tetramer (*P*)-**1** and the (*M*)-pentamer (*M*)-**2** exhibits a drastic reaction shortcut for the formation of hetero-double helix **C** from random coil **A** at 25 °C depending on the thermal pretreatment. It took about 60 h until the reaction was complete *via* the formation of hetero-double helix **B** under the simple-cooling conditions where a solution of random coil **A** at 70 °C was cooled to 25 °C. The **A**-to-**B**-to-**C** reaction was predominant, and the hetero-double helix **B** acted as an off-pathway intermediate, which hampered the **A**-to-**C** reaction. On the other hand, the reaction was complete within 4 h under the freezing–defrosting conditions in which a solution of random coil **A** at 70 °C was snap-cooled to –25 °C before maintaining the solution at 25 °C. The direct **A**-to-**C** reaction was predominant in the latter. The drastic shortcut of the reaction to form **C** from **A** at 25 °C was caused by the thermal pretreatment. The sharp switch of the competing reaction pathways, the **A**-to-**B**-to-**C** or the **A**-to-**C**, occurred because the subtle differences in the initial states at 25 °C generated by the thermal pretreatment were amplified by the self-catalytic process.
