# Supplementary material for: Multiple competing pathways for chemical reaction: drastic reaction shortcut for the self-catalytic double-helix formation of helicene oligomers
Source: Chem Sci. 2016 Oct 14;8(2):1414–21. doi: 10.1039/c6sc01893a (PMC5390785; doi:10.1039/c6sc01893a)
Supplement: Supplementary file 1 [file SC-008-C6SC01893A-s001.pdf]

Electronic Supplementary Material (ESI) for Chemical Science.
This journal is © The Royal Society of Chemistry 2016

Electronic Supplementary Information

Competing Multiple Pathways of Chemical Reaction: Drastic Shortcut of Reaction for Self-Catalytic Double-Helix Formation of Helicene Oligomers

Yo Kushida, Nozomi Saito, Masanori Shigeno, and Masahiko Yamaguchi*

Department of Organic Chemistry, Graduate School of Pharmaceutical Sciences,

Tohoku University, Aoba, Sendai, 980-8578, Japan

*E-mail: yama@m.tohoku.ac.jp, Fax: (+81) 22-795-6811.

Table of contents

General method..........S2
1. Materials..........S2
2. Freezing-defrosting experiments..........S2
3. Simple-cooling experiments..........S3
4. The molecular structures at the initial stage at 25 °C..........S4-S6
5. A-to-C and A-to-B-to-C reaction after cooling at –10 °C S7
6. Experiments at the concentration of 2.5 × 10^{-4} M..........S8-S10
7. Temperature effect in the A-to-C reaction and A-to-B-to-C reaction..........S11-S12
8. Self-catalysis in the A-to-C reaction S13-S14
9. Self-catalysis in the A-to-B reaction..........S15-S17
10. References..........S17

General method. CD and UV-Vis spectra were measured on a JASCO J-720 spectropolarimeter. The path length 0.0107 cm of quartz round cell and 0.0217 cm of quartz square cell were used. Dynamic light scattering (DLS) determined at 173° scattering angle was observed by a Zetasizer Nano S.

1. Materials

(*P*)-**1** and (*M*)-**2** were synthesized as described in our previous work.[S1]

2. Freezing-defrosting experiments

< Sample preparation >

A 1:1 (*P*)-**1**/(*M*)-**2** (total concentration 5.0×10^{-4} M) mixture: (*P*)-**1** (0.70 mg, 2.5×10^{-4} mmol) and (*M*)-**2** (0.84 mg, 2.5×10^{-4} mmol) were dissolved in fluorobenzene (1.0 mL) with heating at 70 °C for 20 min.

< Freezing-defrosting/rapid experiment >

A solution of 1:1 (*P*)-**1**/(*M*)-**2** mixture (total 5.0×10^{-4} M, fluorobenzene) was heated at 70 °C for 30 min, snap cooled at −35 °C by immersing in a cooled bath at –35 °C, transferred to a CD cell adjusted to −25 °C, and allowed to settle for 15 min. The solution was rapidly heated at 25 °C, and allowed to settle for CD analysis (Fig. 3).

< Freezing-defrosting/constant-rate experiment (heating from –25 to 25 °C) at the rate of 2 Kmin^{-1} >

A solution of 1:1 (*P*)-**1**/(*M*)-**2** mixture (total 5.0×10^{-4} M, fluorobenzene) was heated at 70 °C for 30 min, snap cooled at −35 °C by immersing in a cooled bath at –35 °C, transferred to a CD cell adjusted to –25 °C, and allowed to settle for 15 min. The solution was heated from –25 °C to 25 °C at the rate of 2 Kmin^{-1}, and allowed to settle for 120 min for CD analysis (Fig. S1).

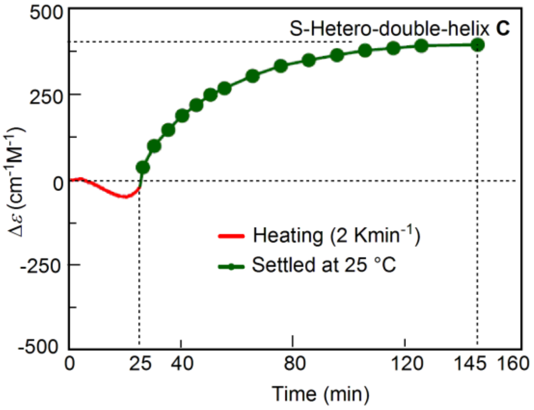

Fig. S1 The Δε (315 nm)/time profile of a 1:1 (*P*)-**1**/(*M*)-**2** mixture in fluorobenzene (total 5.0×10^{-4} M). The solution was heated from –25 °C to 25 °C at the constant-rate of 2 Kmin^{-1} (red line), and settled at 25 °C for 120 min (green circles). The green lines are drawn between points.

3. Simple-cooling experiments

< Simple-cooling/rapid experiment >

A solution of 1:1 (*P*)-**1**/(*M*)-**2** mixture (total 5.0 × 10^{-4} M, fluorobenzene) was first heated at 70 °C for 30 min, rapidly cooled at 25 °C, then allowed to settle for CD analysis.

< Simple-cooling/constant-rate experiment (cooling from 70 to 25 °C) at the rate of 2 Kmin^{-1} >

A solution of 1:1 (*P*)-**1**/(*M*)-**2** mixture (total 5.0 × 10^{-4} M, fluorobenzene) was heated at 70 °C for 30 min. The solution was cooled from 70 °C to 25 °C at the rate of 2 Kmin^{-1}, and allowed to settle for 25 min for CD analysis (Fig. S2).

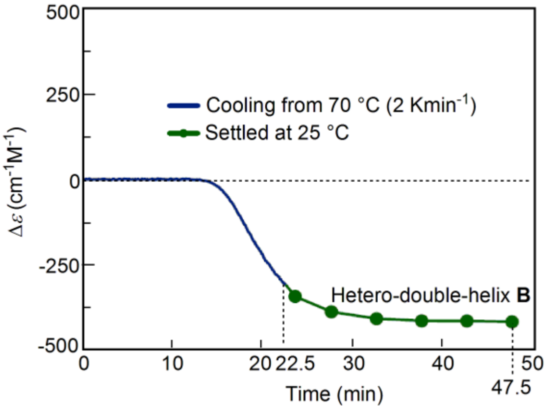

Fig. S2 The Δ*ε* (315 nm)/time profile of a 1:1 (*P*)-**1**/(*M*)-**2** mixture in fluorobenzene (total 5.0 × 10^{-4} M). The solution was cooled from 70 °C to 25 °C at the constant-rate of 2 Kmin^{-1} (blue line), and settled at 25 °C for 25 min (green circles). The green lines are drawn between points.

4. The molecular structures at the initial stage at 25 °C

<< CD Studies >>

< Freezing-defrosting/rapid experiment >

A solution of 1:1 (*P*)-**1**/(*M*)-**2** mixture (total 5.0 × 10^{-4} M, fluorobenzene) was heated at 70 °C for 30 min, snap cooled at −35 °C by immersing in a cooled bath at –35 °C, transferred to a CD cell adjusted to –25 °C, and allowed to settle for 15 min. The solution was rapidly heated at 25 °C and allowed to settle for 20 min, during which CD spectra were obtained at 20 sec intervals until 5 min and at 5 min intervals between 5 and 20 min (Fig. S3).

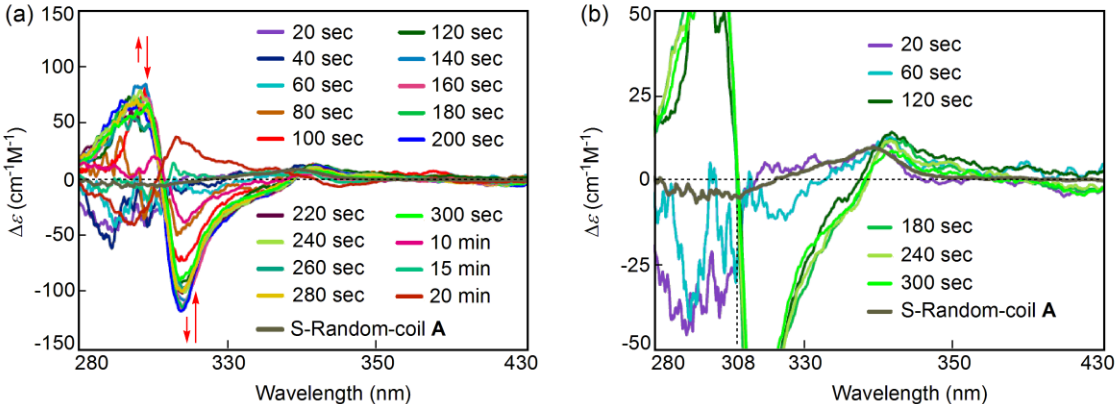

Fig. S3 (a) CD spectra of 1:1 (*P*)-**1**/(*M*)-**2** mixture (total 5.0 × 10^{-4} M, fluorobenzene) showing the initial state in the structural changes from random coil **A** at −25 °C to hetero-double helix **C** at 25 °C under the freezing-defrosting/rapid conditions. CD spectrum of S-random-coil **A** was obtained in our previous work.[S2] (b) Extraction and magnification of (a).

< Simple-cooling/rapid experiment >

A solution of 1:1 (*P*)-**1**/(*M*)-**2** mixture (total 5.0 × 10^{-4} M, fluorobenzene) was heated at 70 °C for 30 min providing an S-random-coil solution. The solution was rapidly cooled at 25 °C then allowed to settle for 5 min, during which CD spectra were obtained at 20 sec intervals (Fig. S4).

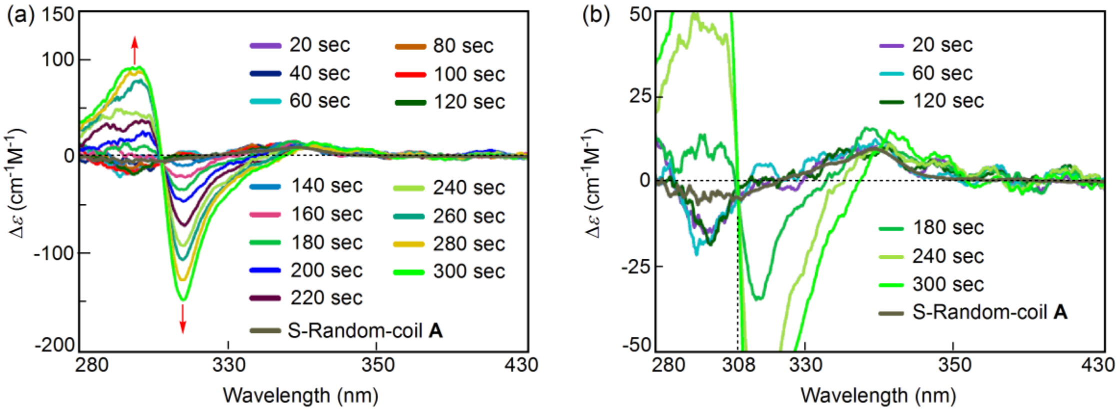

Fig. S4 (a) CD spectra of 1:1 (*P*)-**1**/(*M*)-**2** mixture (total 5.0 × 10^{-4} M, fluorobenzene) showing the initial state in the structural changes from random coil **A** at 70 °C to hetero-double helix **B** at 25 °C under the simple-cooling/rapid conditions. CD spectrum of S-random-coil **A** was obtained in our previous work.[S2] (b) Extraction and magnification of (a).

The spectra in Fig. S3 and S4 were very similar during the initial 300 seconds.

<< **NMR study** >>

< Freezing-defrosting/rapid experiments >

A solution of 1:1 (*P*)-**1**/(*M*)-**2** mixture (total 5.0 × 10^{-4} M, fluorobenzene) was heated at 70 °C for 30 min, snap cooled at −35 °C by immersing in a cooled bath at –35 °C, transferred to a CD cell adjusted to –25 °C, and allowed to settle for 15 min. The solution was rapidly heated at 25 °C, allowed to settle for 2 min, and analyzed by NMR.

<Simple-cooling/rapid experiment >

A solution of 1:1 (*P*)-**1**/(*M*)-**2** mixture (total 5.0 × 10^{-4} M, fluorobenzene) was first heated at 70 °C for 30 min, rapidly cooled at 25 °C, allowed to settle for 2 min, and analyzed by NMR (Fig. S5).

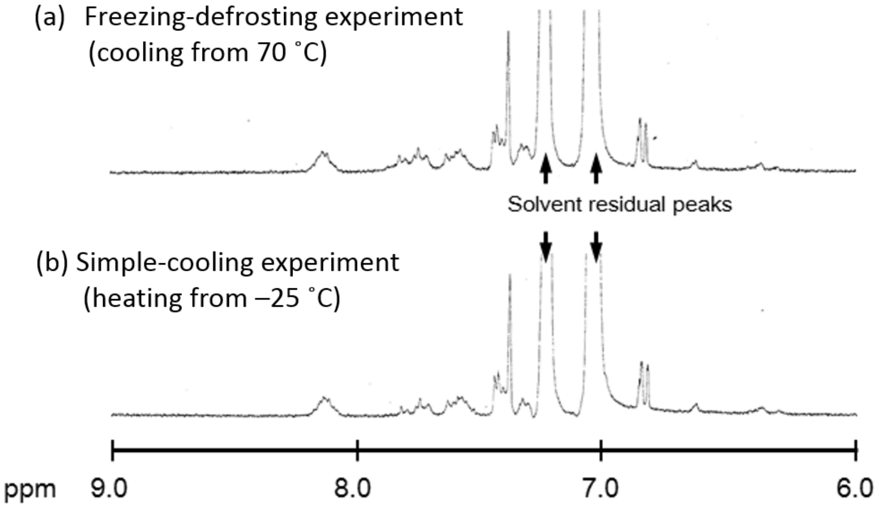

Fig. S5 ^{1}H-NMR spectra of 1:1 (*P*)-**1**/(*M*)-**2** mixture in d_5-fluorobenzene (total 5.0 × 10^{-4} M) in freezing-defrosting and simple-cooling experiments at 25 °C. In the spectra (a) and (b), the solvent residual peaks were adjusted to the same height.

<< **DLS analysis** >>

< **Freezing-defrosting/rapid experiment** >

A solution of 1:1 (*P*)-**1**/(*M*)-**2** mixture (total 5.0 × 10^{-4} M, fluorobenzene) was heated at 70 °C for 30 min, snap cooled at −35 °C by immersing in a cooled bath at –35 °C, transferred to a CD cell adjusted to –25 °C, and allowed to settle for 15 min. The solution was rapidly heated at 25 °C then allowed to settle for 2 min for DLS analysis.

< **Simple-cooling/rapid experiment** >

A solution of 1:1 (*P*)-**1**/(*M*)-**2** mixture (total 5.0 × 10^{-4} M, fluorobenzene) was first heated at 70 °C for 30 min, rapidly cooled at 25 °C, and allowed to settle at that temperature for DLS analysis.

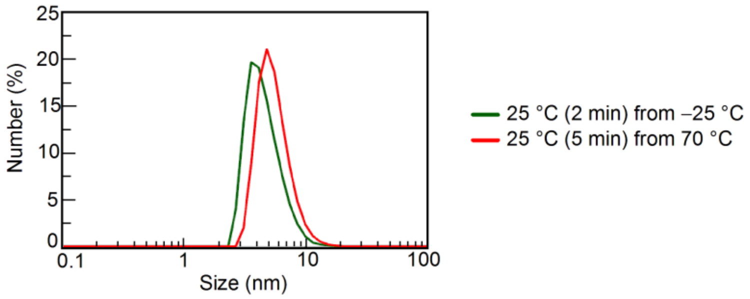

Fig. S6 DLS analysis of 1:1 (*P*)-**1**/(*M*)-**2** mixture in fluorobenzene (total 5.0 × 10^{-4} M) in freezing-defrosting/rapid and simple-cooling/rapid experiments. The average diameters of 5 nm and 6 nm were obtained at 2 min after heating the solution from –25 °C to 25 °C and at 5 min after cooling the solution from 70 °C to 25 °C, respectively.

5. A-to-C and A-to-B-to-C reaction after cooling at –10 °C

< A-to-C reaction: Freezing-defrosting/rapid experiment with snap-cooling at –10 °C >

A solution of 1:1 (*P*)-**1**/(*M*)-**2** mixture (total 5.0 × 10^{-4} M, fluorobenzene) was first heated at 70 °C for 30 min, snap cooled to –35 °C for 15 min by immersing in a cooled bath at –35 °C, transferred to a CD cell adjusted to –10 °C, and allowed to settle for 30 min. Then, the solution was rapidly heated at 25 °C, and allowed to settle for 300 min (Fig. 5, green circles; Fig. S7a).

< A-to-B-to-C reaction: Trapping B by constant-rate cooling to –10 °C at the rate of 2 Kmin^{-1} then rapid-heating experiment >

A solution of 1:1 (*P*)-**1**/(*M*)-**2** mixture (total 5.0 × 10^{-4} M, fluorobenzene) was first heated at 70 °C for 30 min, cooled from 70 °C to −10 °C at the rate of 2 K/min, and allowed to settle for 30 min at −10 °C. Then, the solution was rapidly heated at 25 °C, and allowed to settle for 5040 min (Fig. 5, blue circles; Fig. S7b).

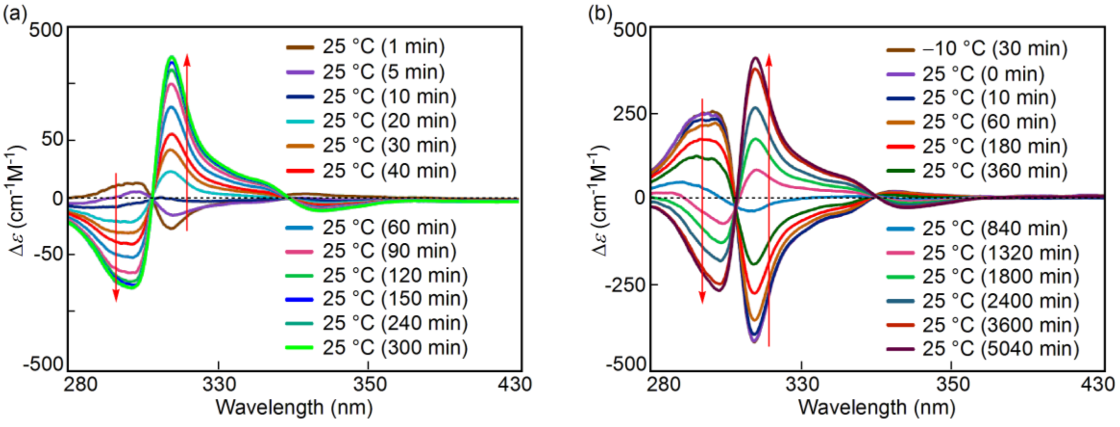

Fig. S7 CD spectra of 1:1 (*P*)-**1**/(*M*)-**2** mixture (total 5.0 × 10^{-4} M, fluorobenzene) showing the structural changes to hetero-double helix **C** at 25 °C. The solution was prepared by (a) freezing-defrosting/rapid method or (b) constant-rate cooling method at the rate of 2 Kmin^{-1}.

6. Experiments at the concentration of 2.5 × 10^{-4} M

< Sample preparation >

A 1:1 (*P*)-**1**/(*M*)-**2** (total concentration 2.5 × 10^{-4} M) mixture: (*P*)-**1** (0.35 mg, 1.25 × 10^{-4} mmol) and (*M*)-**2** (0.420 mg, 1.25 × 10^{-4} mmol) were dissolved in fluorobenzene (1.0 mL) with heating at 70 °C for 20 min.

< Equilibrium states >

A solution of 1:1 (*P*)-**1**/(*M*)-**2** mixture (total 2.5 × 10^{-4} M, fluorobenzene) was heated at 70 °C for 30 min, cooled at 25 °C, and allowed to settle for 86 h providing a solution of hetero-double helix **C**. The solution was cooled at 5 °C for 90 min, 25 °C for 60 min, and 40 °C for 115 min (Fig. S8). At each temperature, the solution was allowed to settle and analyzed by CD for the times described in parentheses (Fig. S8), when $\Delta\varepsilon$ change was less than 2 $cm^{-1}M^{-1}$ within a 10 min period. Then, the equilibrium at 5 °C with $\Delta\varepsilon$ = +410 $cm^{-1}M^{-1}$, 25 °C with $\Delta\varepsilon$ = +360 $cm^{-1}M^{-1}$, and 40 °C with +140 $cm^{-1}M^{-1}$ were determined.

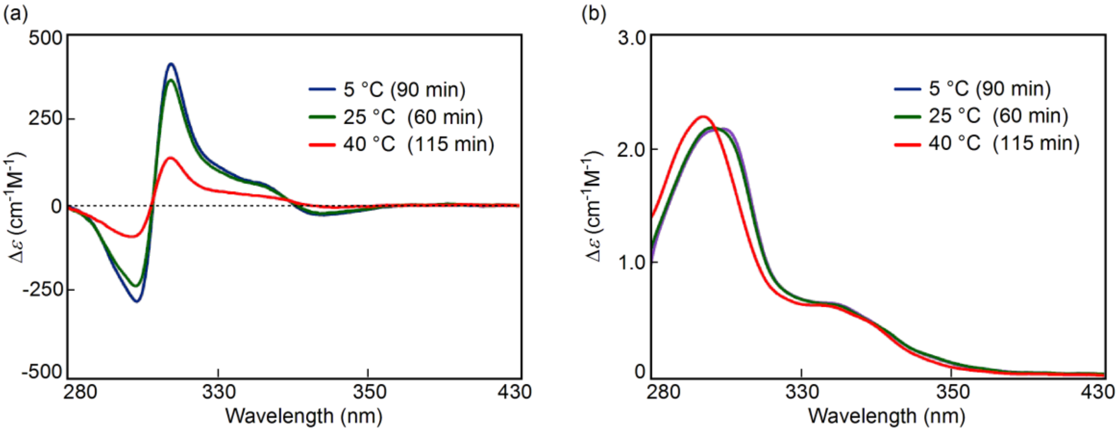

Fig. S8 (a) CD and (b) UV-Vis spectra of 1:1 (*P*)-**1**/(*M*)-**2** mixture in fluorobenzene (total 2.5 × 10^{-4} M) at equilibrium. The spectra were obtained at each temperature after settled for the time shown in parenthesis.

< Freezing-defrosting/rapid experiments >

A solution of 1:1 (*P*)-**1**/(*M*)-**2** mixture (total 2.5×10^{-4} M, fluorobenzene) was heated at 70 °C for 30 min, snap cooled at −35 °C by immersing in a cooled bath at –35 °C, transferred to a CD cell adjusted to –25 °C, and allowed to settle for 15 min. The solution was rapidly heated either at 5 °C, 25 °C, or 40 °C, and allowed to settle for 50-780 min for CD analysis (Fig. S9).

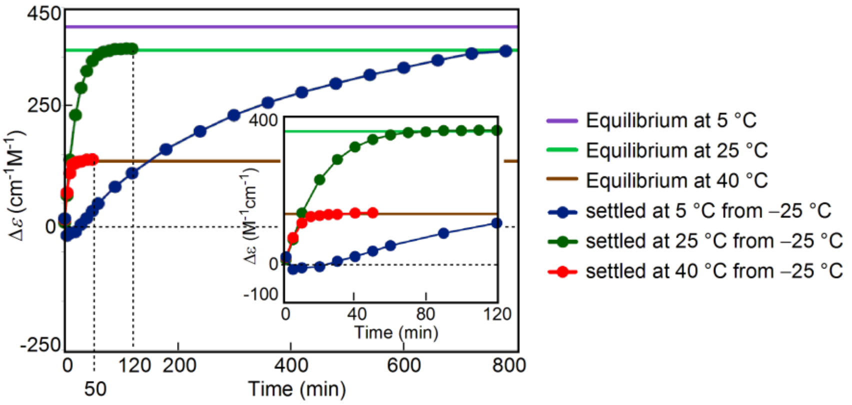

Fig. S9 The Δ*ε* (315 nm)/time profiles of 1:1 (*P*)-**1**/(*M*)-**2** mixtures in fluorobenzene (total 2.5×10^{-4} M) under freezing-defrosting conditions at different temperatures. The profiles at 5, 25, and 40 °C are shown by blue, green, red circles, respectively. The lines are drawn between points. The inset shows the magnification of the profiles between 0 and 120 min. The Δ*ε* values at equilibrium at 5, 25, and 40 °C are shown in purple, pale green, and brown lines, respectively, which were obtained in Fig. S8.

< Simple-cooling/rapid experiments >

A solution of 1:1 (*P*)-**1**/(*M*)-**2** mixture (total 2.5×10^{-4} M, fluorobenzene) was first heated at 70 °C for 30 min, rapidly cooled either at 40, 25, 15, 5, 0, −10, −15, −17.5, or −20 °C, and allowed to settle. The Δ*ε* (315 nm) value at each temperature was followed by CD for 190 min at 40 °C, 40 min between 35 and 5 °C, and 30 min between 0 and −20 °C (Fig. S10). The obtained Δ*ε* values at 20 min were plotted in red circles in Fig. S11.

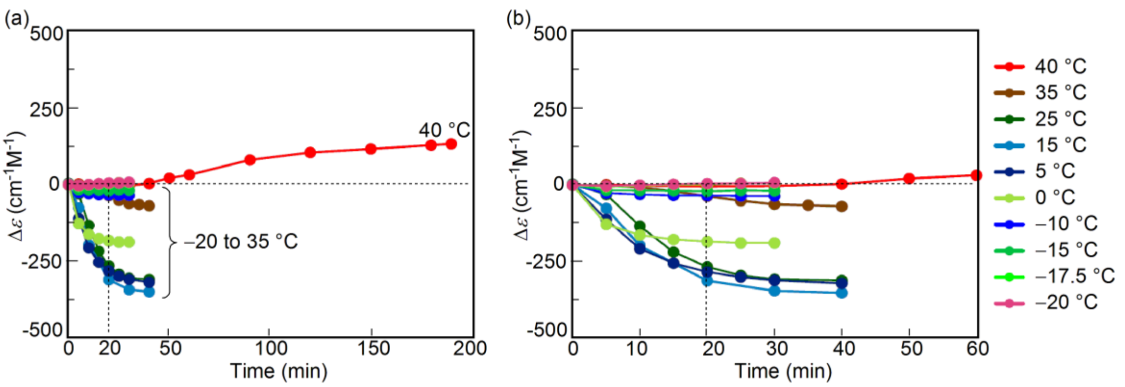

Fig. S10 (a) The Δ*ε* (315 nm)/time profiles of 1:1 (*P*)-**1**/(*M*)-**2** mixtures in fluorobenzene (total 2.5×10^{-4} M) under simple-cooling/rapid conditions. The lines are drawn between points. (b) Magnification

of (a).

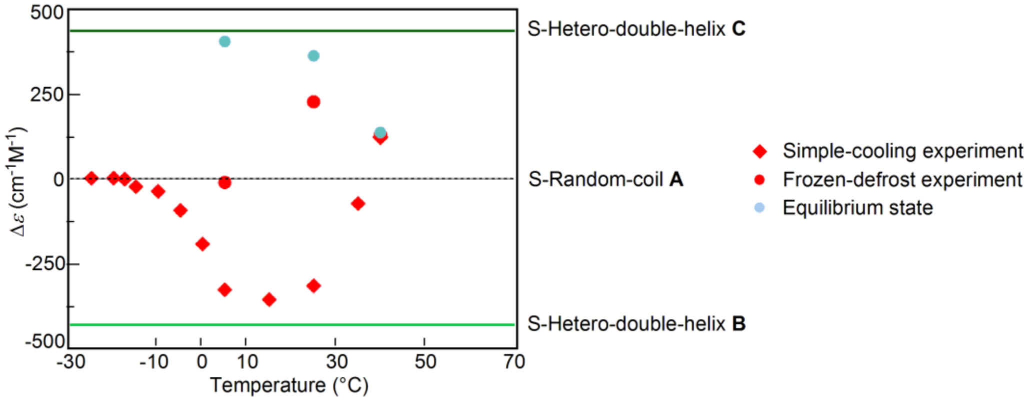

Fig. S11 The Δ*ε* (315 nm)/temperature profiles of 1:1 (*P*)-**1**/(*M*)-**2** mixtures in fluorobenzene (total 2.5 × 10^{-4} M), which were obtained by plotting the Δ*ε* values reached after 20 min periods under the freezing-defrosting/rapid conditions (red circles). The Δ*ε* values obtained under simple-cooling/rapid conditions (red squares) and the values at quilibrium states are also shown (blue circles). The Δ*ε* values of S-random-coil **A**, S-Hetero-double helices **B** and **C**, and at equilibrium between 5 and 50 were obtained in our previous work.[S2]

< Effect of "freezing" temperature >

A solution of 1:1 (*P*)-**1**/(*M*)-**2** mixture (total 2.5 × 10^{-4} M, fluorobenzene) was first heated at 70 °C for 30 min, rapidly cooled at –25, –20, –17.5, or –15 °C, and allowed to settle for 30 min. Then, the solution was rapidly heated at 25 °C and the Δ*ε* values at 315 nm was followed by CD (Fig. S12).

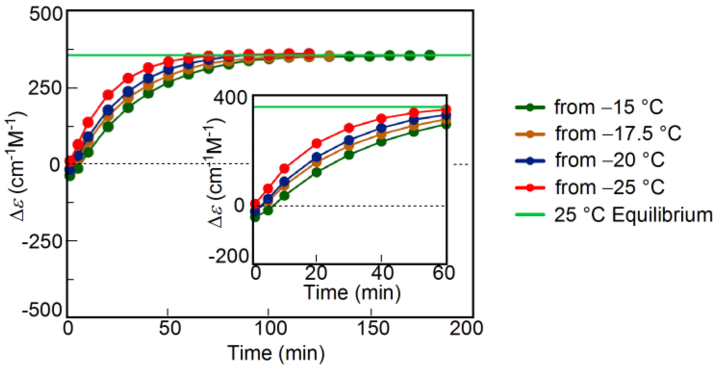

Fig. S12 Time course of the **A**-to-**C** reaction from different "freezing" temperatures obtained by the Δ*ε* (315 nm)/temperature profiles of 1:1 (*P*)-**1**/(*M*)-**2** mixture in fluorobenzene (total 2.5 × 10^{-4} M) at 25 °C. The lines are drawn between points.

7. Temperature effect in the A-to-C reaction and A-to-B-to-C reaction

< Freezing-defrosting/rapid experiment: A-to-C reaction >

A solution of 1:1 (*P*)-**1**/(*M*)-**2** mixture (total 5.0×10^{-4} M, fluorobenzene) was heated at 70 °C for 30 min, snap cooled at −35 °C by immersing in a cooled bath at –35 °C, transferred to a CD cell adjusted to –25 °C, and allowed to settle for 15 min. The solution was rapidly heated either at 5 °C, 25 °C, 40 °C, or 50 °C and allowed to settle for 40-300 min for CD analysis (Fig. S13).

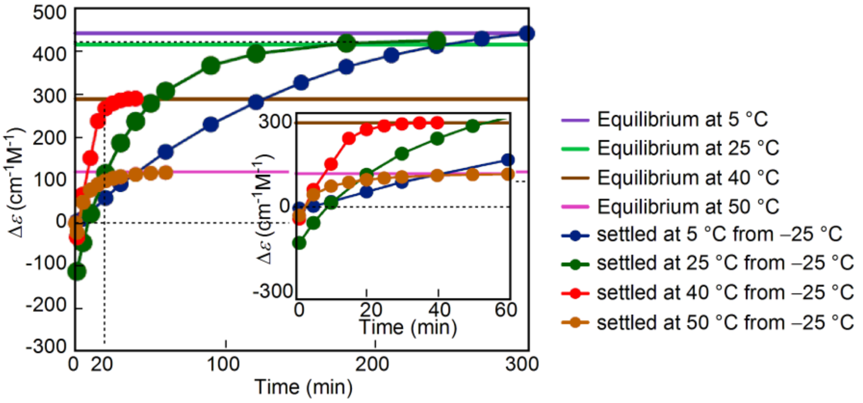

Fig. S13 The Δ*ε* (315 nm)/time profiles of 1:1 (*P*)-**1**/(*M*)-**2** mixtures in fluorobenzene (total 5.0×10^{-4} M) under freezing-defrosting conditions at different temperatures. The profiles at 5, 25, 40, and 50 °C are shown by blue, green, red circles, respectively. The lines are drawn between points. The inset shows the magnification of the profiles between 0 and 60 min. The Δ*ε* values at equilibrium at 5, 25, 40, and 50 °C are shown in purple, pale green, and brown lines, respectively, which were obtained in our previous work.[S2]

< Simple-cooling/rapid experiment: A-to-B-to-C reaction >

A solution of 1:1 (*P*)-**1**/(*M*)-**2** mixture (total 5.0×10^{-4} M, fluorobenzene) was first heated at 70 °C for 30 min, rapidly cooled either at 50, 40, 25, 15, 5, 0, −5, −10, −15, −16, −18, or −20 °C, and allowed to settle. The Δ*ε* (315 nm) value at each temperature was followed by CD for 600 min at 50 °C and 30 min between 40 and −20 °C (Fig. S14). The obtained Δ*ε* values at 315 nm after 20 min were plotted as red circles in Fig. 6.

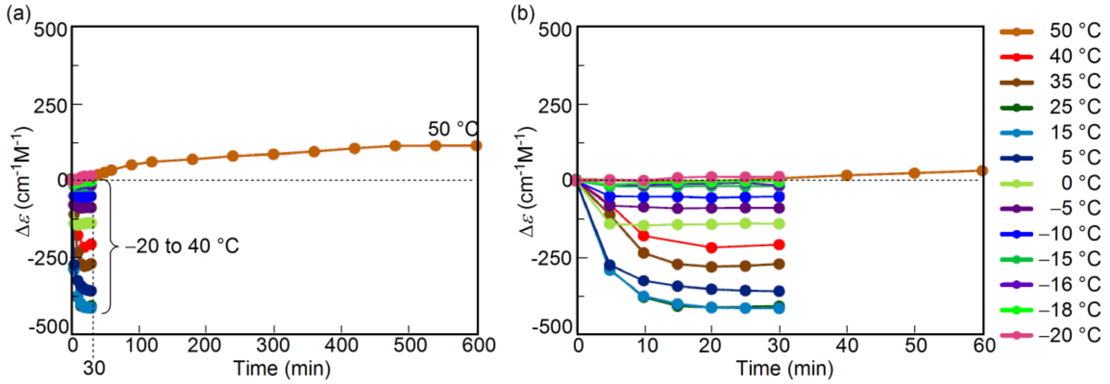

Fig. S14 (a) The Δ*ε* (315 nm)/time profiles of 1:1 (*P*)-**1**/(*M*)-**2** mixtures in fluorobenzene (total 5.0×10^{-4} M) under simple-cooling conditions. The lines are drawn between points. (b) Magnification of (a).

< **Freezing-defrosting/constant-rate experiment** >

A solution of 1:1 (*P*)-**1**/(*M*)-**2** mixture (total 5.0 × 10^{-4} M, fluorobenzene) was first heated at 70 °C for 30 min, snap cooled to –35 °C for 15 min by immersing in a cooled bath at –35 °C, transferred to a CD cell adjusted to –25 °C, and allowed to settle for 15 min. The solution was (1) heated from –25 to 70 °C at the constant-rate of 2 $\mathrm{Kmin^{-1}}$, and then (2) cooled to from 70 to –25 °C at the constant-rate of 2 $\mathrm{Kmin^{-1}}$, during which $\Delta\varepsilon$ values at 315 nm were followed (Fig. 7).

8. Self-catalysis in the A-to-C reaction

< Kinetic analysis >

A solution of 1:1 (*P*)-**1**/(*M*)-**2** mixture (total 5.0 × 10^{-4} M, fluorobenzene) was heated at 70 °C for 30 min, snap cooled at −35 °C by immersing in a cooled bath at –35 °C, transferred to a CD cell adjusted to –25 °C, and allowed to settle for 15 min. The solution was rapidly heated at 5 °C, and allowed to settle for 450 min. The structural change was monitored by the Δ*ε* values at 315 nm (Fig. S15).

Slightly sigmoidal Δ*ε*/time profiles were obtained.

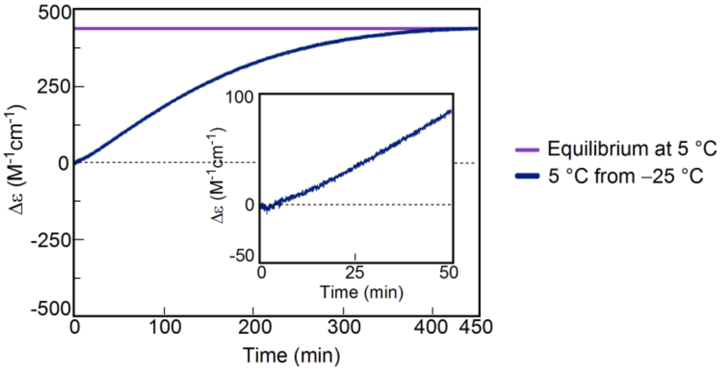

Fig. S15 Δ*ε* (315 nm)/time profiles of 1:1 (*P*)-**1**/(*M*)-**2** mixtures (total 5.0 × 10^{-4} M, fluorobenzene) at 5 °C under freezing-defrosting conditions. The Δ*ε* values of equilibrium was obtained in our previous work.[S2]

< Seeding experiments >

A solution of 1:1 (*P*)-**1**/(*M*)-**2** mixture (total 5.0 × 10^{-4} M, fluorobenzene, 0.60 mL) was transferred to a round quartz cell, heated at 70 °C for 30 min, cooled at 25 °C for 3-4 days, and heated at 40 °C for 100 min providing a solution of hetero-double helix **C** (Fig. S16). Another solution of (*P*)-**1**/(*M*)-**2** mixture (total 5.0 × 10^{-4} M, fluorobenzene, 0.60 mL) was heated at 70 °C for 20 min providing a solution of S-random-coil **A**. The solution of S-random-coil **A** was cooled at 40 °C for 5 sec, and then was added to the solution of hetero-double helix **C** in the quartz cell in a ratio of 1:1 (hetero-double helix **C**:random coil **A** = 0.60 mL:0.60 mL), and the solution was allowed to settle for 240 min for CD analysis (Fig. S17). Experiments in the mixing ratio of 1:2 (hetero-double helix **C**:random coil **A** = 0.40 mL:0.80 mL) and 1:3 (hetero-double helix **C**:random coil **A** = 0.30 mL:0.90 mL) were also conducted (Fig. S17).

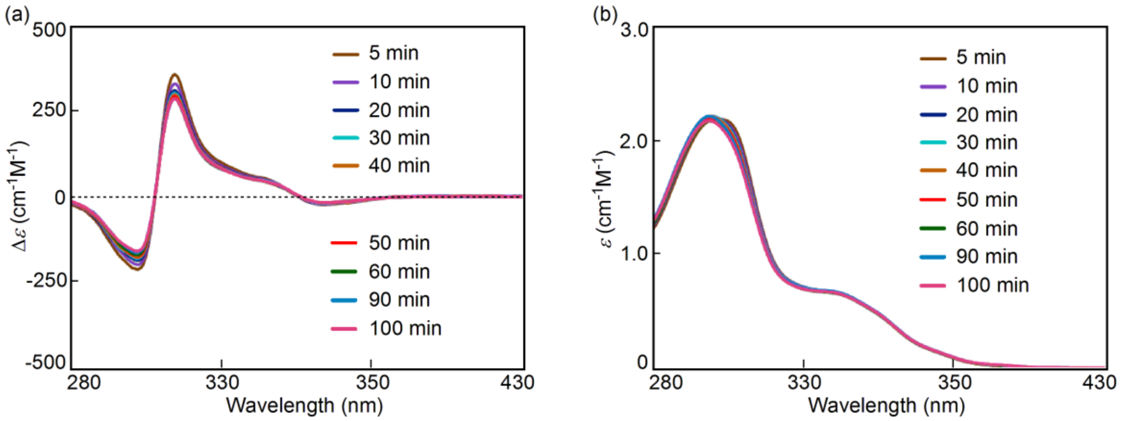

Fig. S16 (a) CD and (b) UV-Vis spectra of (*P*)-**1**/(*M*)-**2** mixture in fluorobenzene (total 5.0 × 10^{-4} M) at

40 °C, which were obtained at each time shown after heating the S-hetero-double-helix solution at 25 °C.[S2]

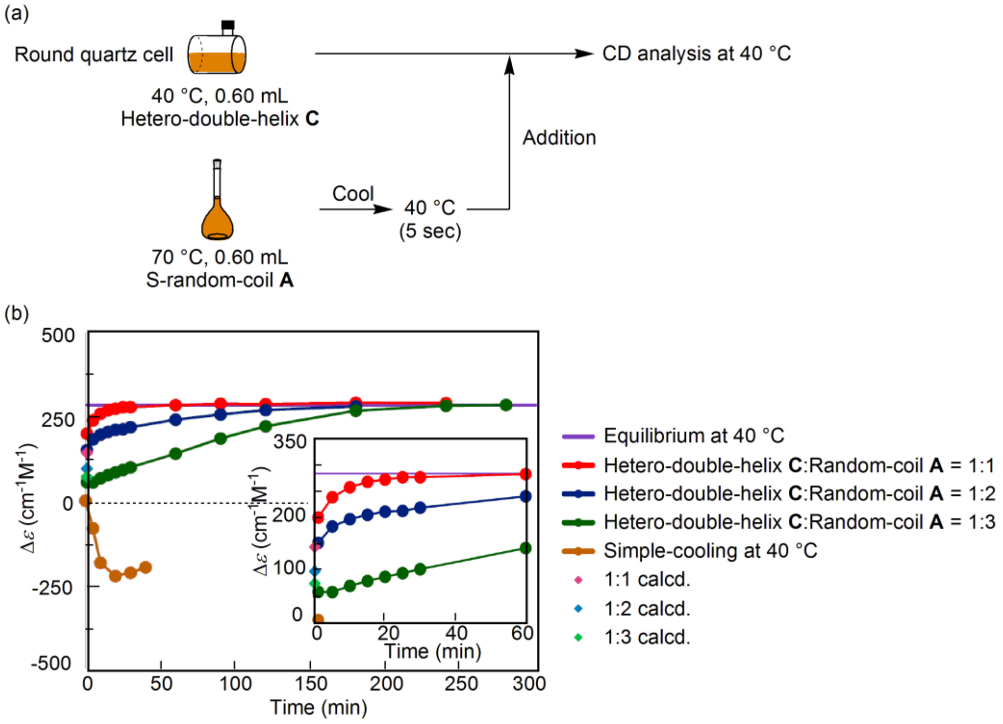

Fig. S17 Seeding experiments for the **A**-to-**C** reaction. (a) A schematic illustration of experimental procedure. (b) Δ*ε* (315 nm)/time profiles of 1:1 (*P*)-**1**/(*M*)-**2** mixture in fluorobenzene (total 5.0 × 10^{-4} M) at 40 °C after an addition of random coil **A**. The inset shows the magnification of the profiles between 0 and 60 min. The circles show Δ*ε* change in the experiments. The pink, pale blue, and pale green squares are calculated value based on the initial ratio of hetero-double helix **C**:random coil **A** = 1:1, 1:2, and 1:3, respectively. The orange circles show Δ*ε* change obtained under simple-cooling conditions at 40 °C (Fig. S13, red circles), where the solution of hetero-double helix **C** is not added. The lines are drawn between points. The Δ*ε* value at equilibrium is shown in a purple line.[S2]

9. Self-catalysis in the A-to-B reaction

< Seeding experiments >

At 45 °C:

A solution of 1:1 (*P*)-**1**/(*M*)-**2** mixture (total 5.0 × 10^{-4} M, fluorobenzene, 0.60 mL) was transferred to a round quartz cell, heated at 70 °C for 20 min, and rapidly cooled at 45 °C for 20 min providing a solution of hetero-double helix **B**. Another solution of (*P*)-**1**/(*M*)-**2** mixture (total 5.0 × 10^{-4} M, fluorobenzene, 0.60 mL) was heated at 70 °C for 20 min providing a solution of S-random-coil **A**. The S-random-coil **A** solution was cooled at 45 °C for 5 sec, and then was added to the solution of hetero-double helix **B** in the quartz cell in a ratio of 1:1 (hetero-double helix **B**:random coil **A** = 0.60 mL:0.60 mL), and the solution was allowed to settle for 20 min for CD analysis (Fig. S18b).

At 25 °C:

A solution of 1:1 (*P*)-**1**/(*M*)-**2** mixture (total 5.0 × 10^{-4} M, fluorobenzene, 0.60 mL) was transferred to a round quartz cell, heated at 70 °C for 20 min, and rapidly cooled at 25 °C for 20 min providing a solution of hetero-double helix **B**. Another solution of (*P*)-**1**/(*M*)-**2** mixture (total 5.0 × 10^{-4} M, fluorobenzene, 0.60 mL) was heated at 70 °C for 20 min providing a solution of S-random-coil **A**. The S-random-coil **A** solution was cooled at 25 °C for 5 sec, and then was added to the solution of hetero-double helix **B** in the quartz cell in a ratio of 1:1 (hetero-double helix **B**:random coil **A** = 0.60 mL:0.60 mL), and the solution was allowed to settle for 20 min for CD analysis (Fig. S18c).

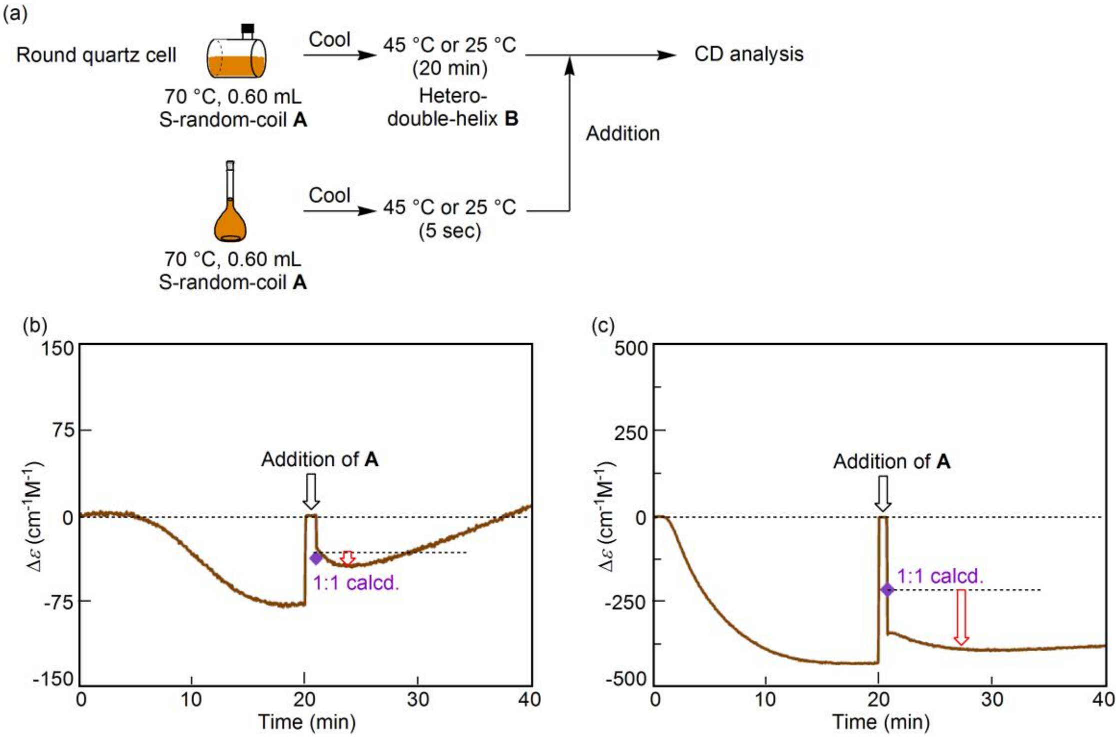

Fig. S18 Seeding experiments for the **A**-to-**B** reaction shown (a) A schematic illustration of experimental procedure. Δ*ε* (315 nm)/time profiles of 1:1 (*P*)-**1**/(*M*)-**2** mixtures in fluorobenzene (total 5.0 × 10^{-4} M) at (b) 45 °C and (c) 25 °C. The brown curves show Δ*ε* change in the experiments. The purple squares are calculated values based on the ratio of hetero-double helix **B**:random coil **A** = 1:1. Increase and decrease in Δ*ε* values on addition of random coil **A** are due to removal and setting of quartz cell from CD instrument. The initial increases of hetero-double helix **B** are shown by the downward red arrows from the calculated Δ*ε* values.

< **Control experiments** >

At 45 °C and 25 °C:

A solution of 1:1 (*P*)-**1**/(*M*)-**2** mixture (total 5.0 × 10^{-4} M, fluorobenzene, 1.2 mL) was transferred to a round quartz cell, heated at 70 °C for 20 min, and rapidly cooled at 45 °C (Fig. S19a) or 25 °C (Fig. S19b) for 40 min. Compare Fig. S17b with S18a, and also S17c with S18b: No decrease of $\Delta\varepsilon$ as shown by the red arrows appeared in this control experiments.

Addition of solvent (fluorobenzene) at 25 °C:

A solution of 1:1 (*P*)-**1**/(*M*)-**2** mixture (total 5.0 × 10^{-4} M, fluorobenzene, 0.60 mL) was transferred to a round quartz cell, heated at 70 °C for 20 min, and cooled at 25 °C for 20 min providing a solution of hetero-double helix **B**. Fluorobenzene was added to the hetero-double helix **B** solution in the quartz cell in a ratio of 1:1 (hetero-double helix **B**:fluorobenzene = 0.60 mL:0.60 mL), and the solution was allowed to settle for 20 min for CD analysis (Fig. S19d). Compare with Fig. S18c.

Addition of hetero-double helix B to hetero-double helix B at 25 °C:

A solution of 1:1 (*P*)-**1**/(*M*)-**2** mixture (total 5.0 × 10^{-4} M, fluorobenzene, 0.60 mL) was transferred to a round quartz cell, heated at 70 °C for 20 min, and cooled at 25 °C for 20 min providing a solution of hetero-double-helix **B**. Another solution of (*P*)-**1**/(*M*)-**2** mixture (total 5.0 × 10^{-4} M, fluorobenzene, 0.60 mL) was heated at 70 °C for 20 min, and cooled at 25 °C for 20 min providing a solution of hetero-double helix **B**, which was added to the hetero-double helix **B** solution in the quartz cell in a ratio of 1:1 (hetero-double helix **B**:hetero-double helix **B** = 0.60 mL:0.60 mL), and the solution was allowed to settle for 20 min for CD analysis (Fig. S19e). No decrease of $\Delta\varepsilon$ was observed in this control experiment. Compare with Fig. S18c.

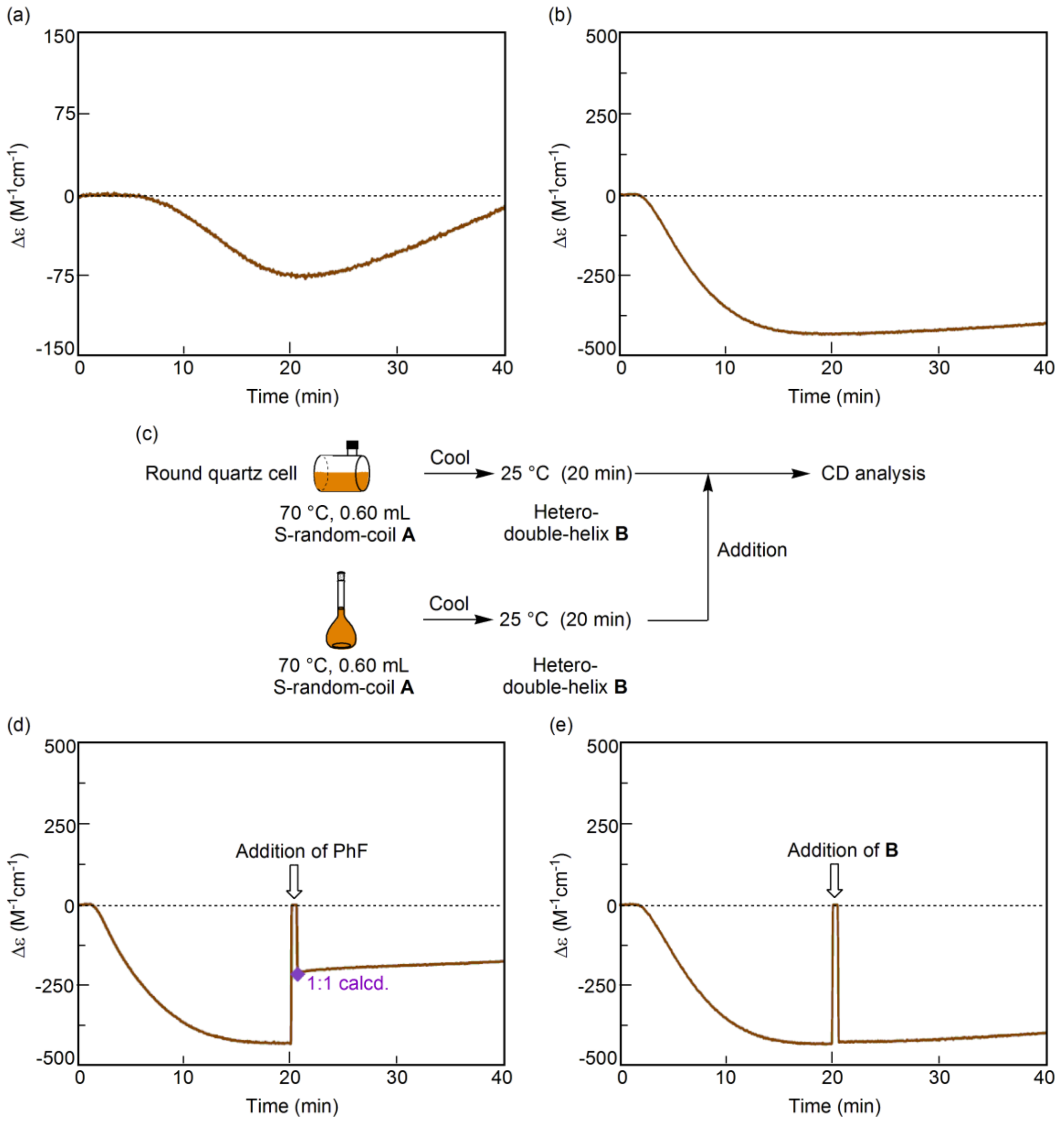

Fig. S19 Control experiments for the **A**-to-**B** reaction shown by Δε (315 nm)/time profiles of (*P*)-**1**/(*M*)-**2** mixtures in fluorobenzene (total 5.0×10^{-4} M) with simple-cooling from 70 °C to (a) 45 °C and (b) 25 °C and with an addition of (d) fluorobenzene or (e) hetero-double helix **B** at 25 °C. (c) A schematic illustration of the experimental procedure in Fig. S19e.

10. References

(S1) Shigeno, M.; Sato, M.; Kushida, Y.; Yamaguchi, M. *Asian J. Org. Chem.*, **2014**, *3*, 797.

(S2) Shigeno, M.; Kushida, Y.; Yamaguchi, M. *J. Am. Chem. Soc.*, **2014**, *136*, 7972.
